# Effects of social media sponsorship disclosure on adolescents’ advertising literacy and purchase intention

**DOI:** 10.1371/journal.pone.0348505

**Published:** 2026-05-27

**Authors:** Ya-En Catherine Lin, Fong-Ching Chang, Tzu-Fu Huang, Shawn C. Chiang, Chiung-Hui Chiu, Ping-Hung Chen, Nae-Fang Miao, Hung-Yi Chuang

**Affiliations:** 1 Department of Health Promotion and Health Education, National Taiwan Normal University, Taipei, Taiwan; 2 Shin Kong Wu Ho-Su Memorial Hospital, Taipei, Taiwan; 3 Department of Health Behavior, School of Public Health, Texas A&M Health Science Center, College Station, Texas, United States of America; 4 Graduate Institute of Information and Computer Education, National Taiwan Normal University, Taipei, Taiwan; 5 Graduate Institute of Mass Communication, National Taiwan Normal University, Taipei, Taiwan; 6 Post-Baccalaureate Program in Nursing, College of Nursing, Taipei Medical University, Taipei, Taiwan; 7 Department of Public Health, Kaohsiung Medical University, Kaohsiung, Taiwan; UMass Amherst: University of Massachusetts Amherst, UNITED STATES OF AMERICA

## Abstract

**Introduction:**

In the rapidly evolving digital age, adolescents have become a particularly vulnerable group impacted by social media marketing. This study aims to examine how different types of sponsorship disclosure (full, partial, and none) in social media affect adolescents’ ability to recognize advertising, their advertising literacy, and their purchase intentions.

**Materials and methods:**

A self-administered questionnaire was used to assess the effects of three types of sponsorship disclosure in social media posts on adolescents. In 2022, a total of 3,149 high school students were recruited from 36 schools across Taiwan using a probability proportional to size sampling method. Adolescents were randomly assigned to one of three experimental groups and exposed to social media posts featuring varying levels of sponsorship disclosure.

**Results:**

The study found that adolescents exposed to full sponsorship disclosure had significantly higher rates of advertisement recognition and greater conceptual advertising literacy compared to those in the partial or no disclosure conditions. The multivariate analysis revealed that advertisement recognition was associated with higher levels of both conceptual and attitudinal advertising literacy. Adolescents who could recognize advertisements demonstrated higher attitudinal advertising literacy, which was associated with lower purchase intentions.

**Conclusions:**

Clear sponsorship disclosure is crucial for raising advertising awareness among adolescents, enhancing their advertising literacy, and potentially protecting them from increased susceptibility to purchase intentions.

## Introduction

As children and adolescents spend increasing amounts of time on social media, consuming digital influencer content, digital advertising has emerged as a powerful force shaping their lives [1,2]. Unlike traditional advertising, influencer marketing is subtle and difficult to identify because marketers blur the boundaries between paid and organic content [2]. This blurred distinction can lead to severe negative impacts on young people’s physical and mental health [3]. Recent scoping evidence highlights concerns about health impacts of exposure to influencer marketing across multiple domains [4–8]. For instance, exposure to influencer marketing on social media has been shown to increase the risk of addictive substance use, such as alcohol, tobacco, e-cigarettes, and problem gambling behaviors, among children and adolescents [4]. Adolescents who actively follow multiple influencers report higher levels of social appearance anxiety and social media addiction [5]. Within the digital food environment specifically, children and adolescents face near-ubiquitous exposure to food marketing on social media—much of it promoting ultra-processed foods [6]. A review of 51 studies found that influencer content frequently promotes unrealistic body ideals and unhealthy products to adolescents [7]. Despite this growing evidence, critical research gaps remain. Most research has been conducted in the Global North, and existing studies lack consistent methodological approaches [7]. Moreover, voluntary industry self-regulation has proven largely ineffective in the online environment, highlighting the need for government regulation and improved digital advertising literacy among youth [6].

Social media influencer marketing can blur the line between personal content and paid ads, often making sponsored posts indistinguishable from genuine recommendations [9–11]. In response to this challenge, government entities have issued guidelines on when and how to disclose sponsored social media posts with the goal to enhance transparency, prevent confusion, and safeguard consumers from misleading and deceptive practices. In the United States, the Federal Trade Commission (FTC) has established disclosure requirements, while the United Kingdom’s Advertising Standards Authority (ASA) has implemented similar guidelines [12–16]. These guidelines recommend the use of clear, unambiguous language and caution against abbreviations or vague terms, though enforcement and platform implementation vary significantly [17]. The UK maintains stricter guidelines, with the ASA and European Advertising Standards Alliance (EASA) mandating explicit labeling such as #ad, showing some evidence of better compliance and improved youth advertising recognition [17–19]. Beyond the frequently cited guidelines from the United States and the United Kingdom, other jurisdictions have begun addressing the regulation of influencer marketing and sponsorship disclosures. Several European countries, along with Australia, China, Japan, and South Korea markets, have introduced advertising standards and consumer protection laws that extend to digital and influencer content [16,20]. However, most of these frameworks remain fragmented, unevenly enforced, and often lack specific provisions tailored to adolescents, creating both research and policy gaps.

Social media platforms like Instagram and TikTok have implemented disclosure tools that label branded content as advertising [21–23]. Research shows these tools can help youth recognize advertising, especially when disclosures appear early in posts and are presented clearly [21]. However, labeling content as advertising alone may be insufficient to alter youth attitudes or behaviors, highlighting the need for ongoing regulatory refinement and complementary educational interventions [21–23]. In practice, disclosure formats vary considerably. The most common approach involves only hashtags such as #ad or #sponsored placed at the end of posts, which would be considered as partial disclosure. Approaches that incorporate multiple transparency mechanisms simultaneously, such as explicit verbal statements (e.g., “This post is sponsored by @brandname”), brand tags, and multiple hashtags identifying both the advertisement and brand, would be considered full disclosure, and such disclosure aligns with the comprehensive regulatory recommendations from bodies like the FTC and ASA [12,14,15,24]. The distinction between these approaches is critical, as research suggests that multi-element disclosures may be more effective at activating persuasion knowledge than hashtags alone.

Despite these evolving platform practices, the situation remains concerning in regions with limited regulatory frameworks: there are no or limited regulations for influencer marketing disclosures in majority of nations, including Taiwan. Even where such regulation exists, implementation challenges persist. Studies have shown that most people remain unable to distinguish between influencer recommendations and paid promotions on social media [9,10]. This persistent lack of transparency, even in regulated environments, highlights how vulnerable consumers- particularly children and younger audiences- remain to the often-undetectable influence of paid digital promotions.

Significant gaps remain in our understanding of adolescent health risks associated with influencer marketing, [6,7,25,26]. Most research has been conducted in Western contexts, leaving Asian high-income countries like Taiwan underrepresented. Additionally, older adolescents, children from minority ethnic or lower-income families, and youth outside high-income countries remain understudied. The age group of 16–18 year olds is particularly underrepresented despite being at critical developmental risk. Research consistently shows that as children age, their advertising literacy and critical reasoning improve, but these abilities develop gradually and are not fully developed even in adolescence [25,26]. A 2022 systematic review found that critical reasoning abilities are not fully developed by age 16, and adolescents remain susceptible to effects of advertising, especially in digital environments [25]. The review explicitly called for more primary research focused on teenagers, particularly those aged 16–18, as this age group is underrepresented in current studies despite being highly exposed to complex digital marketing.

### Theoretical framework

The Persuasion Knowledge Model (PKM) remains a core model for understanding how consumers recognize, interpret and respond to sponsorship disclosures in influencer marketing. PKM suggests that consumers develop knowledge about persuasion tactics over time, which enables them to identify persuasive intent and activate cognitive defenses against marketing messages [27]. Recent studies explicitly use PKM to examine how disclosure (e.g., #ad, #sponsored) activates advertising recognition, which mediates the effects of influencer credibility and parasocial interaction on purchase intentions, especially among millennials and social media users [28–30]. While originally developed for adult, subsequent studies have extended PKM to children and adolescents, suggesting that their developing persuasion knowledge is a crucial factor in determining how disclosures work in this age group [31–34]. In the context of adolescents, PKM implies that clearer disclosures should facilitate recognition of commercial intent and stimulate the activation of persuasion knowledge, which in turn shapes how young audiences evaluate and respond to influencer content [35].

Within the PKM-based approach, there are two complementary facets of advertising literacy [36,37]. First, conceptual advertising literacy refers to adolescents’ knowledge-oriented understanding that a post is commercial and persuasive (e.g., recognizing sponsorship, identifying selling intent, and noticing persuasive tactics). Second, attitudinal advertising literacy captures their evaluative stance toward persuasion (e.g., skepticism, critical orientation, and willingness to resist). These facets align with PKM’s expectation that activated persuasion knowledge via sponsorship disclosure can manifest both as greater awareness (conceptual) and as defensive evaluations (attitudinal), each with distinct implications for outcomes such as purchase intention [27,38].

Recent literature examining sponsorship disclosure effects on adolescents’ responses to influencer marketing has primarily centered on the PKM, investigating both conceptual and attitudinal dimensions of advertising literacy. Studies consistently demonstrate that sponsorship disclosures activate conceptual persuasion knowledge—adolescents’ recognition of advertising and understanding of persuasive intent [17,39,40]. Platform-specific research reveals important contextual variations: Instagram native advertisements posing as social posts yield effects comparable to user-generated content despite disclosure presence [41], while TikTok disclosures successfully activate persuasion knowledge without mitigating brand attitudes or product choice [21,40]. Source credibility emerges as a critical moderating factor, with disclosure effects varying significantly between celebrity versus brand sources [9,42].

Despite the presence of sponsorship disclosure information in influencer marketing, studies have revealed that over half of consumers fail to notice such disclosures [9,41], which limits their effectiveness in activating persuasion knowledge. While several studies have explored different formats of sponsorship disclosure in influencer marketing, the majority have focused on maximizing commercial gains rather than enhancing media literacy [39,42]. The current study examines how different disclosure types (e.g., full, partial, or no disclosure; wording, placement, or prominence) differentially activate conceptual versus attitudinal advertising literacy in adolescents—and in turn, shape downstream outcomes such as purchase intentions. By systematically varying disclosure type, our study addresses this gap and clarifies when disclosure cues move beyond mere recognition to meaningful resistance or, alternatively, to inferences of authenticity that sustain persuasion.

### Research hypotheses

Grounded in the Persuasion Knowledge Model (PKM) and prior empirical findings, this study tests the effects of different sponsorship disclosure types (full, partial, none) on adolescents’ ability to recognize advertising, advertising literacy, and their purchase intentions.

**H1.** Full sponsorship disclosure group will have higher ad recognition compared to partial or no disclosure groups.

**H2.** Full sponsorship disclosure group will have higher advertising literacy (conceptual, attitudinal) compared to partial or no disclosure groups.

**H3.** Full sponsorship disclosure group will have lower purchase intention than partial or no disclosure groups.

**H4.** Adolescents who recognize the ad will report higher advertising literacy (conceptual, attitudinal).

**H5.** Adolescents with higher advertising literacy will have lower purchase intention.

The findings of this study will not only fill a critical research gap but also provide policymakers with evidence-based recommendations for establishing clear and effective standards for sponsorship disclosure.

## Materials and methods

### Participants

Adolescents (N = 3,149) were recruited from 36 schools in urban and rural areas in Taiwan in 2022. A probability proportionate to size sampling method was used to systematically draw a random sample of schools. Four to six classes in each of the sample schools were selected. The sampling frame was a list of schools and high-school student enrollments from the Ministry of Education 2021 academic year data.

### Procedure

Due to restrictions related to the COVID-19 pandemic at the time of the study, unaffiliated individuals were not permitted to enter schools. Consequently, the research team reached out to the teachers responsible for student health at each participating school to explain the study and enlist their assistance in administering the survey. Written consent was given to both students and their parents, and teachers were asked to explain the study orally to ensure comprehension. The school-based survey was conducted from April 25 to June 30, 2022. Teachers were asked to introduce the survey to students, distribute consent forms and questionnaires, and oversee the process within classrooms. In total, 3,251 high school students (ages 15–18) completed the self-administered questionnaires. After excluding questionnaires with missing data, 3,149 responses were deemed valid (boys: 1,500; girls: 1,649). The response rate was approximately 89%.

A self-administered questionnaire was used to conduct the experiment. Adolescents were randomly assigned to one of the three experimental conditions, full disclosure, partial disclosure, and no disclosure (control). Each experimental condition presented an identical social media post depicting a casual indoor setting where four young people were gathered around a table. The post featured a can of *Heineken 0.0* (zero-alcohol beer) placed prominently in the center of the table as the focal point, with the individuals appearing to interact socially in what resembled a typical friend gathering scenario. The accompanying text was written in Mandarin Chinese and described a birthday celebration where the poster brought *Heineken 0.0* to share with friends, commenting on its taste (similar to dark malt beverage but with hop and malt flavors like regular beer) and noting that zero-alcohol beer prevents hangovers. The post encouraged friends to drink it together at future gatherings.

The three experimental conditions differed only in their disclosure elements: In the full disclosure condition, the first sentence of the post clearly stated, “This post is sponsored by @Heineken,” accompanied by a tag marker for the brand. Additionally, clear hashtags were included at the end of the post, such as “#Ad,” “#BrandName,” and “#ProductName” (in both English and Chinese). In the partial disclosure condition, only hashtags were placed at the end of the post, with no disclosure text included. In the no disclosure condition, neither text nor hashtags were provided. Adolescents were invited to view posts (which included text and a photo) that varied by experimental type. After viewing the post, they answered a series of questions regarding their perceptions of the post (e.g., “Is the post an ad or not an ad?”), as well as their understanding, attitude toward the post, and intention to purchase the product mentioned.

### Materials

The questionnaire was developed on the basis of previous research [10,27,36,37,43–49]. The content validity was assessed by a group of six experts. A pretest survey was conducted to interpret the adolescents’ responses to the survey and to evaluate the reliability of the scales in the questionnaire. Adolescents were assured that their information would be protected and anonymous. Approval was obtained from the Institutional Review Board at National Taiwan Normal University (202012HS010).

### Measures

#### Ad recognition.

Ad recognition and social media advertising literacy were adapted from the Persuasion Knowledge Scale of Sponsored Content (PKS-SC) and the Children’s Advertising Literacy Scale [37,43]. Ad recognition was assessed with a single item. After viewing the social media post, adolescents were asked to respond to the ad recognition measure, which read, “Do you think this post is an ad or commercial?” with answer options of “Yes” or “No.”

#### Advertising literacy.

Based on previous research [27,36,37,45,47], advertising literacy was measured by assessing two dimensions: conceptual and attitudinal advertising literacy. Conceptual advertising literacy in the present study was measured with four questions: “This post is to stimulate people to want the product,” “The brand pays for showing the product in this post,” “The brand tries to influence me by making sure this post does not look like advertising,” and “The brand tries to influence me by placing the brand in a context that I like”). The scale ranged from 1 (strongly disagree) to 4 (strongly agree), with higher score indicating higher conceptual advertising literacy. Attitudinal advertising literacy in the present study was measured with four items: “I think showing brands in this post is not trustworthy,” “I think it is dishonest if the post does not disclose the sponsoring brand,” “While reading, I countered the information in the post,” and “I feel disgusted if the post does not indicate advertising or sponsorship upfront.” These items were also rated on a 4-point scale from 1 (strongly disagree) to 4 (strongly agree), with higher score indicating higher attitudinal advertising literacy.

#### Purchase intention.

The intention to purchase the product from the post was measured using two items, based on prior research [10,44,46]. Adolescents were asked, “How likely are you to buy the product from the post?” and “How likely are you to try the product from the post?”. Response options for each item were rated on a 4-point scale from 1 to 4, including “definitely not”, “probably not”, “probably yes”, and “definitely yes”.

#### Demographic variables.

Demographic information includes gender (male vs. female) and grade (10th grade, 11th grade, 12th grade).

### Data analysis

All analyses were conducted using SPSS version 23.0 (Armonk, NY: IBM Corp.). Percentages and means were calculated for all variables. Chi-squared tests were conducted to analyze gender, grade, and ad recognition differences in three experimental types. ANOVA was conducted to analyze effects of disclosure types on advertising literacy and purchase intention. Multiple logistic regression and multiple regression were conducted to examine the factors related to ad recognition, advertising literacy and purchase intention.

Before testing the structural relationships, we examined the psychometric properties of all latent constructs. A confirmatory factor analysis (CFA) was conducted using AMOS 27 with maximum likelihood estimation to assess the measurement model. Item-level reliability was evaluated by inspecting factor loadings, with values above .50 considered acceptable. Internal consistency was assessed using Cronbach’s α, while composite reliability (CR) was calculated to capture the overall reliability of each construct. Convergent validity was examined by calculating the average variance extracted (AVE), with values above .50 indicating adequate convergence. Discriminant validity was evaluated using both the Fornell–Larcker criterion. The results of these measurement checks are reported in Tables 2 and 3.

## Results

### Adolescents’ characteristics and ad recognition

Among the 3,149 adolescents, 1,039 (33%) were in the full disclosure group, 1,036 (33%) were in the partial disclosure group, and 1,074 (34%) were in the no disclosure group. There were no significant differences in gender or grade distribution across the three groups. The Chi-squared test revealed significant differences in adolescents’ ability to recognize ads across the disclosure groups. Adolescents in the full disclosure group recognized ads (80%) more effectively than those in the partial disclosure group (69%) and the no disclosure group (65%) (Table 1). These results support H1, indicating that full sponsorship disclosure significantly enhanced adolescents’ recognition of advertising content compared with partial or no disclosure.

**Table 1:** Demographic variables and ad recognition across three disclosure types.

	No Disclosure		Partial Disclosure		Full Disclosure		P value
	n	%	n	%	n	%		
Gender							.449
Female	565	52.6	527	50.9	557	53.6	
Male	509	47.4	509	49.1	482	46.4	
Grade							.922
10th	451	42.0	422	40.7	416	40.0	
11th	363	33.8	362	34.9	365	35.1	
12th	160	24.2	252	24.3	258	24.8	
Ad recognition							<.001
No	376	35.0	317	30.6	208	20.0	
Yes	698	65.0	719	69.4	831	80.0	

Note. Chi-squared tests were conducted. N = 3149 (No disclosure n = 1074, Partial disclosure n = 1036, Full disclosure n = 1039).

### Adolescents’ advertising literacy and purchase intention

Before testing the hypothesized relationships, we first evaluated the measurement model to establish the reliability and validity of the study constructs; the results of these assessments are summarized in Tables 2 and 3. Table 2 presents the descriptive statistics, factor loadings, and reliability/validity indices for the three study constructs. For conceptual advertising literacy, item means ranged from 2.36 to 2.83, with factor loadings between .472 and .686. Cronbach’s α was .681, composite reliability (CR) was .669, and the average variance extracted (AVE) was .341, indicating marginal internal consistency and weak convergent validity. For attitudinal advertising literacy, item means ranged from 2.25 to 2.45, with factor loadings between .501 and .794. The construct demonstrated acceptable reliability (α = .756, CR = .756), although the AVE (.456) fell slightly below the recommended threshold. For purchase intention, the two items showed means (1.91–2.06) and strong factor loadings (.822–.887). Reliability and validity indices were excellent (α = .843, CR = .843, AVE = .732). Overall, these results suggest that while purchase intention and attitudinal advertising literacy were measured reliably.

**Table 2:** Item descriptive, factor loadings, and construct reliability/validity.

Items	Mean	SD	Loading Factor (λ)	Cronbach’s Alpha	Composite Reliability	Average Variance Extracted (AVE)
Conceptual advertising literacy				0.681	0.669	0.341
“This post is to stimulate people to want the product.”	2.44	0.77	0.602			
“The brand pays for showing the product in this post.”	2.83	0.86	0.472			
“The brand tries to influence me by making sure this post does not look like advertising.”	2.48	0.85	0.551			
“The brand tries to influence me by placing the brand in a context that I like.”	2.36	0.87	0.686			
Attitudinal advertising literacy				0.756	0.756	0.456
“I think showing brands in this post is not trustworthy.”	2.25	0.77	0.591			
“I think it is dishonest if the post does not disclose the sponsoring brand.”	2.45	0.79	0.501			
“While reading, I countered the information in the post.”	2.34	0.79	0.772			
“I feel disgusted if the post does not indicate advertising or sponsorship upfront.”	2.28	0.81	0.794			
Purchase Intention				0.843	0.843	0.732
“How likely are you to buy the product from the post?”	2.06	0.82	0.887			
“How likely are you to try the product from the post?”	1.91	0.77	0.822			

**Table 3:** Discriminant validity (Fornell–Larcker matrix) and construct correlations.

	Conceptual advertising literacy	Attitudinal advertising literacy	Purchase intention
Conceptual advertising literacy	0.583		
Attitudinal advertising literacy	0.452	0.675	
Purchase intention	0.467	0.180	0.856

Discriminant validity was examined using the Fornell–Larcker criterion. As shown in Table 3, the square root of AVE for each construct (conceptual advertising literacy = .583, attitudinal advertising literacy = .675, purchase intention = .856) was greater than the correlations with other constructs, satisfying the Fornell–Larcker criterion.

### Effects of disclosure types on ad recognition, advertising literacy, and purchase intention

Adolescents in the full disclosure group demonstrated higher levels of conceptual advertising literacy compared to those in the partial or no disclosure groups. However, there were no significant differences in adolescents’ attitudinal advertising literacy or purchase intentions across the three disclosure types (Table 4). These findings provide partial support for H2. While full disclosure appears to strengthen adolescents’ understanding of the persuasive and commercial intent behind influencer content (conceptual advertising literacy), it did not significantly alter their evaluative or critical attitudes toward such advertising (attitudinal advertising literacy).

**Table 4:** Effects of disclosure types on advertising literacy and purchase intention.

	No Disclosure		Partial Disclosure		Full Disclosure		P value	Post-hoc tests
	Mean	SD	Mean	SD	Mean	SD		
Conceptual advertising literacy	2.47	0.60	2.53	0.60	2.59	0.60	<.001***	F > P F > N
Attitudinal advertising literacy	2.34	0.62	2.33	0.60	2.32	0.58	.808	
Purchase intention	1.96	0.73	2.00	0.76	1.99	0.73	.290	

Note. ANOVA was conducted. Post-hoc tests were analyzed by Scheffe test. N = 3136 (F = Full disclosure; P = Partial disclosure; and N = No disclosure).

After controlling for gender and grade, multiple logistic regression analysis indicated that adolescents in the full or partial disclosure groups compared with no disclosure group were more likely to recognize advertisements. In addition, multiple regression showed that adolescents in the full disclosure group also had higher levels of conceptual advertising literacy. There were no significant differences in attitudinal advertising literacy and purchase intention among the three disclosure types (Table 5). Thus, H3 was not supported. Although full sponsorship disclosure enhanced ad recognition and conceptual advertising literacy, it did not lead to a reduction in purchase intention.

**Table 5:** Effects of disclosure types on ad recognition, literacies, and purchase intention.

	Ad recognition			Conceptual advertising literacy			Attitudinal advertising literacy			Purchase intention		
	β	SE	p	β	SE	p	β	SE	p	β	SE	p
Intercept	0.70	0.09	<.001	2.49	0.02	<.001	2.35	0.02	<.001	1.90	0.03	<.001
Gender												
Male vs. Female	−0.28	0.08	0.01	−0.02	0.02	0.381	−0.01	0.02	0.506	0.13	0.03	<.001
Grade												
11^th^ grade vs. 10^th^ grade	0.14	0.09	0.139	−0.01	0.03	0.654	−0.03	0.03	0.141	−0.04	0.03	0.255
12^th^ grade vs. 10^th^ grade	0.03	0.10	0.806	−0.03	0.03	0.255	0.01	0.03	0.906	0.03	0.03	0.454
Disclosure type												
Partial vs. No disclosure	0.20	0.09	0.028	0.06	0.03	0.023	−0.01	0.03	0.887	0.05	0.03	0.145
Full vs. No disclosure	0.70	0.09	<.001	0.13	0.03	<.001	−0.02	0.03	0.536	0.04	0.03	0.236

Note. Multiple logistic analysis was conducted for ad recognition model (n = 3149). Multiple regression analysis was conducted for conceptual advertising literacy (n = 3136), attitudinal advertising literacy (n = 3137), and purchase intention (n = 3144).

### Relationships between ad recognition, advertising literacy, and purchase intention

The results of the multiple regression analysis indicated that, after controlling for gender and grade, adolescents who recognized ads in the posts were more likely to have higher levels of both conceptual and attitudinal advertising literacy. Additionally, adolescents in the full disclosure group were more likely to exhibit higher levels of conceptual advertising literacy. Furthermore, adolescents who recognized advertisements, had higher levels of attitudinal advertising literacy and lower levels of conceptual advertising literacy, showed higher purchase intentions (Table 6). These results support H4, indicating that ad recognition is positively associated with both conceptual and attitudinal dimensions of advertising literacy. For H5, the regression model predicting purchase intention revealed a more complex pattern. Ad recognition and attitudinal advertising literacy were negatively associated with purchase intention, whereas conceptual advertising literacy showed a positive association with purchase intention. Thus, H5 was only partially supported. While higher attitudinal advertising literacy appeared to reduce purchase intention as expected, greater conceptual advertising literacy was unexpectedly linked with increased purchase intention.

**Table 6:** Relationships between ad recognition, advertising literacy, and purchase intention.

	Conceptual advertising literacy			Attitudinal advertising literacy			Purchase intention		
	β	SE	p	β	SE	p	β	SE	p
Intercept	2.20	0.03	<.001	2.24	0.03	<.001	0.53	0.06	<.001
Gender									
Male vs. Female	0.01	0.02	0.785	−0.01	−0.02	0.803	0.13	0.02	<.001
Grade									
11^th^ grade vs. 10^th^ grade	−0.02	0.02	0.334	−0.04	0.03	0.113	−0.02	0.03	0.477
12^th^ grade vs. 10^th^ grade	−0.03	0.03	0.198	0.00	0.03	0.883	0.05	0.03	0.113
Disclosure type									
Partial vs. No disclosure	−0.04	0.03	0.115	−0.01	0.03	0.664	0.01	0.03	0.618
Full vs. No disclosure	0.06	0.03	0.014	−0.04	0.03	0.127	−0.03	0.03	0.385
Ad recognition									
Yes vs. No	0.44	0.02	<.001	0.16	0.02	<.001	−0.16	0.03	<.001
Conceptual advertising literacy							0.65	0.02	<.001
Attitudinal advertising literacy							−0.06	0.02	0.005

Note. Multiple regression was conducted for conceptual advertising literacy (n = 3136), attitudinal advertising literacy (n = 3125) and purchase intention (n = 3121).

## Discussion

This study examined how different types of sponsorship disclosures in social media posts affect adolescents’ ability to recognize advertising, their advertising literacy, and purchase intentions. The findings revealed that full sponsorship disclosures significantly improved ad recognition compared to partial or no disclosures. This aligns with previous studies [9,17,50–53], showing that clear, upfront disclosures strongly influence adolescents’ perception of posts as advertising. Regulatory bodies such as the U.S. Federal Trade Commission (FTC), the U.K.’s Advertising Standards Authority (ASA), the European Aviation Safety Agency (EASA), and Japan’s Act against Unjustifiable Premiums have established guidelines to improve transparency and protect consumers from deceptive marketing [12,14,24]. These guidelines emphasize clear, explicit language and discourage ambiguous terminology. Countries with limited or no current regulations, such as Taiwan, could adopt similar measures to safeguard adolescents from misleading influencer marketing.

This study found that while full disclosure improved ad recognition and conceptual advertising literacy, it did not significantly impact adolescents’ attitudinal advertising literacy toward digital advertising or their purchase intentions. This suggests that simply disclosing sponsorship may not be sufficient to influence adolescents’ attitudes toward advertising or their likelihood of buying a product. This finding aligns with previous studies demonstrating that while disclosures consistently activate conceptual persuasion knowledge and recognition [17,39,40,54,55], this cognitive activation does not necessarily translate into attitudinal defenses or altered behavioral outcomes [34,48]. However, this cognitive activation does not necessarily translate into attitudinal defenses or altered behavioral outcomes. For instance, while disclosures increase ad recognition, they often fail to generate critical skepticism [34,48] or may even produce paradoxical positive effects on purchase intentions through perceived transparency and authenticity [32,41]. Previous studies similarly found that disclosures may fail to generate critical skepticism [34,48] or even produce paradoxical positive effects through perceived transparency [32,41]. Furthermore, a study found that participants who viewed sponsorship influencer posts alongside an advertising literacy intervention were more likely to activate persuasion knowledge and attenuate their purchase intentions compared to participants who only received sponsorship disclosure without the advertising intervention [56]. This pattern suggests that disclosure alone may be insufficient, and that platform context and source characteristics play important moderating roles in how adolescents process persuasive content [9,40–42]. It appears that the recognition of advertising triggers a cognitive process that enhances adolescents’ attention to the content, leading to increased memory retention, but may not initiate an affective process that influences their evaluations of the brand. Therefore, media literacy interventions beyond disclosure are crucial for adolescents to protect them from the influences of social media marketing.

Furthermore, this study found that the relationship between advertising literacy and purchase intention varied by literacy type. Attitudinal advertising literacy was negatively associated with purchase intention, suggesting that adolescents who hold more critical views about advertising and its potential for manipulation are less likely to be swayed toward purchasing the advertised products. Consistent with previous studies [56], this finding reinforces the importance of advertising literacy in empowering young consumers to make informed choices. This finding also aligns with earlier research highlighting the role of critical thinking, resistance coping strategies, and media literacy in mitigating the persuasive effects of advertising [47]. However, conceptual advertising literacy, counterintuitively, was positively related to purchase intention. A previous study suggested that children are unlikely to activate and use their conceptual advertising knowledge as critical advertising defense due to their immature sociocognitive skills and the affect-based nature of contemporary advertising which causes them primarily process advertising on a low elaborative and affective level [36]. This indicates that an understanding of advertising does not make adolescents immune to its persuasive appeals; instead, they may be more drawn to cleverly crafted messages or campaigns that resonate with their interests. Therefore, as previous studies have suggested children and adolescents might need attitudinal advertising literacy, which includes low-effort, attitudinal mechanisms, that can be effective in reducing children’s advertising susceptibility under conditions of low elaboration [36]. Recent literature also emphasizes the need for tailored media literacy interventions for older adolescents and highlights the lack of research specifically targeting this age group [26]. Beyond the impacts on health, influencer marketing have also shown to influence youth purchasing behaviors via increased trust, perceived friendliness/rapport, and authenticity of influencer endorsements [57]. Together, these findings highlight the need for expanded research in under-studied contexts and improved monitoring of harmful marketing practices on social platforms.

The observed effects in this study are specific to older adolescents (ages 15–18), who occupy a transitional developmental stage between childhood and adulthood. At this stage, adolescents possess a more sophisticated but still maturing understanding of persuasive intent. This may explain why full sponsorship disclosures enhanced ad recognition and conceptual advertising literacy but did not translate into changes in attitudes or purchase intentions. In contrast, younger children whose cognitive and persuasion knowledge systems are less developed would likely show weaker recognition effects and be more easily influenced by sponsored content. Adults, on the other hand, typically demonstrate stronger skepticism toward advertising and greater resistance to persuasive appeals. Accordingly, the present findings and their implications are most relevant for adolescent-focused media policies and educational programs, emphasizing the need to strengthen advertising literacy training and transparency standards specifically for this age group.

## Conclusions

This study examined the impact of different sponsorship disclosure types on adolescents’ ability to recognize advertising, their social media advertising literacy, and their purchase intentions. The findings indicate that full sponsorship disclosure significantly improves adolescents’ ability to recognize advertisements and enhances their conceptual advertising literacy. This suggests that clear, unambiguous disclosures help adolescents better understand the persuasive intent behind influencer content. While full disclosure improves ad recognition and conceptual advertising literacy, it does not significantly influence adolescents’ attitudes toward advertising or their purchase intentions. This highlights that awareness alone may not reduce the desire to engage with advertised products. Additionally, the study found that both ad recognition and attitudinal advertising literacy may serve as protective factors against heightened purchase intentions among adolescents. Therefore, effective disclosure guidelines and media literacy education are essential to empower young consumers to recognize ads, critically evaluate social media marketing, and resist its influence.

### Limitations

This study has several limitations. First, it primarily focused on the effects of three specific disclosure formats: full disclosure, partial disclosure using hashtags, and no disclosure. It did not explore the potential influence of other variations in disclosure language, placement, or prominence, which could elicit different responses from adolescents. Further research investigating a wider array of disclosure variations is necessary to gain a more comprehensive understanding of how adolescents perceive and respond to different approaches to transparency in influencer marketing. Second, the findings of this study are based on a single product category (zero-alcohol beer). The persuasive appeal of influencer marketing and the effectiveness of disclosures may vary significantly depending on the product being promoted. Products that adolescents perceive as high-involvement or personally relevant may be less affected by disclosures compared to those they view as low-risk or less important. Future research should examine whether these findings generalize across different product categories to determine if certain products are more susceptible to the influence of sponsorship disclosures than others. Third, as the study specifically targeted adolescents, the results may not generalize to younger children or adults. Developmental differences in cognitive capacity, media literacy, and advertising skepticism could lead to distinct responses across age groups. Fourth, while purchase intention and attitudinal advertising literacy demonstrated satisfactory psychometric properties, conceptual advertising literacy showed weaker measurement performance (AVE < .50), suggesting adolescents may inconsistently recognize persuasive intent. Future research should refine measurement items for this construct to improve validity. Future studies should explore how disclosure effects vary by developmental stage to clarify whether adolescents’ responses are uniquely sensitive to sponsorship transparency. Despite these limitations, the use of a large sample of adolescents and rigorous study design in this study further facilitated the understanding of the effects of social media sponsorship disclosure on adolescents.

### Theoretical and practical implications

This study extends existing theories of advertising literacy and the persuasion knowledge model (PKM) by demonstrating that different types of sponsorship disclosures in social media posts activate distinct cognitive and attitudinal processes among adolescents. While full sponsorship disclosure significantly enhanced ad recognition and conceptual advertising literacy, it did not translate into changes in attitudinal advertising literacy or purchase intention. This pattern suggests that recognition of persuasive intent engages adolescents’ cognitive understanding of advertising but may not elicit affective resistance to persuasive messages, a finding consistent with prior research [17,58,59]. This disconnect between cognitive recognition and behavioral outcomes suggests that advertising literacy alone may be insufficient to alter purchase intentions. Purchase intentions are shaped by multiple interacting factors beyond disclosure and literacy, including influencer credibility, parasocial relationships, content quality, peer and parental influence, and perceived brand fit [58–60]. These findings may underscore the need for theoretical models that account for the multifaceted nature of persuasion in digital environments and the limitations of disclosure-based interventions in isolation.

From a practical perspective, the findings offer several insights for policymakers, educators, and social media practitioners. First, regulatory agencies should ensure that sponsorship disclosures are clear, explicit, and placed prominently within influencer content to enhance recognition among young audiences. Second, as disclosure alone does not appear sufficient to alter attitudes or behavior, these regulatory efforts should be complemented by media-literacy programs in schools that foster adolescents’ critical and attitudinal advertising literacy. Educator and non-profit organizations can integrate influencer-marketing examples into classroom discussions to help students recognize and evaluate persuasive tactics. Third, brands and influencers should adopt transparent communication practices and consider the ethical implications of marketing to underage audiences. Together, these measures can help balance commercial interests with consumer protection, promoting a more responsible and transparent digital advertising environment.
